# Retrieving Soil Physical Properties by Assimilating SMAP Brightness Temperature Observations into the Community Land Model

**DOI:** 10.3390/s23052620

**Published:** 2023-02-27

**Authors:** Hong Zhao, Yijian Zeng, Xujun Han, Zhongbo Su

**Affiliations:** 1 Faculty of Geo-Information Science and Earth Observation (ITC), University of Twente, Hengelosestraat 99, 7514 AE Enschede, The Netherlands; 2 Chongqing Engineering Research Center for Remote Sensing Big Data Application, School of Geographical Sciences, Southwest University, Chongqing 400715, China; 3 Key Laboratory of Subsurface Hydrology and Ecological Effect in Arid Region of Ministry of Education, School of Water and Environment, Chang’an University, Xi’an 710054, China

**Keywords:** soil properties, data assimilation, unified passive and active microwave observation operator, CLM, SMAP, brightness temperature, uncertainties

## Abstract

This paper coupled a unified passive and active microwave observation operator—namely, an enhanced, physically-based, discrete emission-scattering model—with the community land model (CLM) in a data assimilation (DA) system. By implementing the system default local ensemble transform Kalman filter (LETKF) algorithm, the Soil Moisture Active and Passive (SMAP) brightness temperature $T_{B}^{p}$ (p = Horizontal or Vertical polarization) assimilations for only soil property retrieval and both soil properties and soil moisture estimates were investigated with the aid of in situ observations at the Maqu site. The results indicate improved estimates of soil properties of the topmost layer in comparison to measurements, as well as of the profile. Specifically, both assimilations of $T_{B}^{H}$ lead to over a 48% reduction in root mean square errors (RMSEs) for the retrieved clay fraction from the background compared to the top layer measurements. Both assimilations of $T_{B}^{V}$ reduce RMSEs by 36% for the sand fraction and by 28% for the clay fraction. However, the DA estimated soil moisture and land surface fluxes still exhibit discrepancies when compared to the measurements. The retrieved accurate soil properties alone are inadequate to improve those estimates. The discussed uncertainties (e.g., fixed PTF structures) in the CLM model structures should be mitigated.

## 1. Introduction

Soil moisture (SM) is a key variable in the Earth system linking the global water, energy and carbon cycles [1] and impacting the climate system through main processes, such as the partitioning of incoming radiation to the latent and heat fluxes and boundary layer stability [2,3]. SM strongly influences hydrological and agricultural processes, particularly in the semi-arid and arid regions, where strong coupling between SM and precipitation occurs [4,5], and vegetation (and associated carbon uptake) is more sensitive to soil water availability [6,7]. Spatiotemporally consistent SM information can be obtained using land surface models (LSMs) by assimilating in situ and remote sensing observations [8,9,10,11,12], and recent reviews of this state-of-the-art can be found in [13,14].

In LSMs, the Richard equation with the specification of soil hydraulic properties (i.e., soil water retention characteristic and hydraulic conductivity, denoted as SHPs) [15] is used to model soil water flow processes [16,17,18]. Parameters in SHP functions are estimated through pedotransfer functions (PTFs) [19,20], which employ basic soil properties (i.e., soil texture and organic matter content) data extracted from the existing global soil datasets (e.g., Harmonized World Soil Database [21]) as the input. This consideration from basic soil properties to SHPs guarantees soil physical consistency in the land–atmosphere process. However, the uncertainties (e.g., limited in situ soil profiles, the uncertainty in input variable and covariates and interpolation accuracy) in soil property datasets might cause biases in predicted SHPs, and hence, introduce uncertainties in representing the land surface states by LSMs [22,23,24].

To obtain basic soil properties and associated SHPs at the large scale (e.g., km scale of LSMs), the data assimilation (DA) strategy combining LSMs and observations has been investigated [25,26,27]. Satellite data involving thermal emission, namely, brightness temperature ($T_{B}^{p}$, with p = Horizontal or Vertical polarization) measured with passive microwave sensors and retrieved SM from passive, active and combined passive–active sensor products (i.e., $T_{B}^{p}$ and backscattering coefficient $\sigma^{0}$), are the two main direct assimilation quantities to LSMs [14]. Soil moisture data assimilation (SM DA) is widely used to infer soil texture and hydraulic properties [28,29,30,31]. Recent studies even adopted SM DA to calibrate parameters of pedotransfer functions (PTFs) for improving SM estimation with LSM [32,33]. While current satellite SM products contain considerable biases, especially in the high-latitude regions [22,34,35,36,37], due to the uncertainties in the specified parameters (e.g., surface roughness and vegetation optical depth) in the passive microwave remote sensing SM retrieval model [38,39]. These foregoing uncertainties can propagate to the retrieved soil physical properties [28,40].

Moreover, in SM retrieval, the current computation of the soil dielectric constant relies on semi-empirical formulations based on measurements over specific soil samples [41,42]. Generally, soil texture information (i.e., percentages of sand and clay) is one of the inputs. This background information used in the retrieval may deviate from that used in LSMs, and the same situation occurs in the soil temperature and vegetation information used in SM retrieval. Furthermore, the soil structure related to organic matter does affect soil hydraulic and dielectric properties [23,43,44,45]; this aspect still undergoes the process of explicit investigation and refinement [39,46]. To approach a dynamic Earth-observation-based soil (physical property) monitoring system with physical consistency, including a microwave radiative transfer model (RTM) in the DA system deserves to be explored, which is also reported to be attractive for coupled land–atmosphere DA considering its physical consistency [47].

Dedicated studies have employed the zeroth-order RTM (also called the tau-omega model for passive remote sensing) to retrieve soil properties by assimilating $T_{B}^{p}$ [48,49,50]. While it is known that many empirical assumptions were made in the tau-omega model, for instance, the site-specific best-fit approach was applied to obtain surface roughness parameters, similar site-specific empirical equations with the inputting of the vegetation index were used to estimate vegetation optical depth (tau) [39]. This application also introduces unexpected uncertainties to soil property retrieval. Furthermore, research has shown successful estimates of SM at a finer scale (e.g., meter) using active satellite radar (backscatter $\sigma^{0}$) observations. With the existing and upcoming launched satellite missions, such as the NASA-ISRO L-band SAR (NISAR, planned launch date in 2023) [51] and ESA High Priority Candidate Mission Radar Observation System for Europe in L-band (ROSE-L, planned launch date in 2027) [52], it offers a great opportunity to retrieve soil properties at the fine scale. It is noted that active and passive microwave sensor products exhibit different sensitivities to soil and vegetation parameters and provide complementary information on the observed scene [53,54]. Including a unified passive and active microwave observation operator in the DA system is of significance in enhancing the understanding of how best to use the existing and future satellite microwave observations for improving the representation of soil physical properties and land surface states and fluxes with LSM in a physically consistent manner.

Many efforts have been made to develop the physically-based scattering-emission model through Maxwell’s equations under the support of the complementary relationship between the emission and scattering [55]. The examples are an integral equation model (IEM) [56] and its advanced version (AIEM) [57] for a rough, bare soil surface, and a discrete scattering model (notably the Tor Vergata model) [58] for a vegetated surface. It is known that AIEM assumes isotropic roughness properties for soil half-space and does not account for the dielectric effects due to heterogeneities in the soil medium (e.g., composition, moisture content and bulk density). A physically-based surface dielectric roughness model named an air-to-soil transition (ATS) model [59] has been developed to account for this and further integrated with the coupled AIEM to TVG model (thereafter ATS-AIEM-TVG) for modeling L-band scattering and emission of the overall vegetation-soil medium.

In light of the above, the first objective of this paper is to include the aforementioned integrated ATS-AIEM-TVG (abbreviated as TVG) model with enhanced physical considerations as a unified passive and active microwave observation operator in a land DA system. This kind of physically-based microwave observation operator coupling is for the first time to the best of our knowledge. Specifically, the TVG is coupled with the CLM version 4.5 [60] (hereafter, CLM) in an open-source, multivariate, land data assimilation system (called DasPy), which is easy to use due to software availability and adaptability [61,62,63]. Based on the developed DA system, the second objective of this paper is to investigate and answer whether the passive Soil Moisture Active and Passive (SMAP) $T_{B}^{p}$ assimilation improves estimates of basic soil properties and their vertical descriptions, and if so, to investigate and answer whether the refined soil property characterization improves estimates of SM and soil temperature profiles with the CLM, as well as land surface fluxes. As the soil particle structure (i.e., platy or sphere) differs within soil texture [15], the corresponding soil moisture content may induce differences in the observed $T_{B}^{H}$ and $T_{B}^{V}$ [64], and, in turn, the retrieval using either $T_{B}^{H}$ or $T_{B}^{V}$ may apply for different soil particle fraction estimates. Few studies investigate this aspect. Thus, by conducting brightness temperature at horizontal polarization ($T_{B}^{H}$) or brightness temperature at vertical polarization ($T_{B}^{V}$) assimilations, the third objective of this study is to investigate and answer whether the retrieved soil property is polarization-dependent. The Maqu site (33.91° N, 102.16° E) on the eastern Tibetan Plateau providing comprehensive field observations [34,36,37,38] is selected as the study area for the aforementioned investigations. The SMAP Level-1C (L1C) $T_{B}^{P}$ product is assimilated through the DasPy’s default DA algorithm of the local ensemble transform Kalman filter (LETKF) [65].

The paper is organized as follows. Section 2 presents the Maqu site observations, a brief description of the DA system developed in this case (i.e., the CLM, the TVG model, the LETKF algorithm and $T_{B}^{p}$ observations) and the experimental design that served for the aforementioned investigations. The results and discussions are provided in Section 3. Conclusions are drawn in Section 4.

## 2. Materials and Methods

### 2.1. Maqu Site Observations

**Figure 1:**
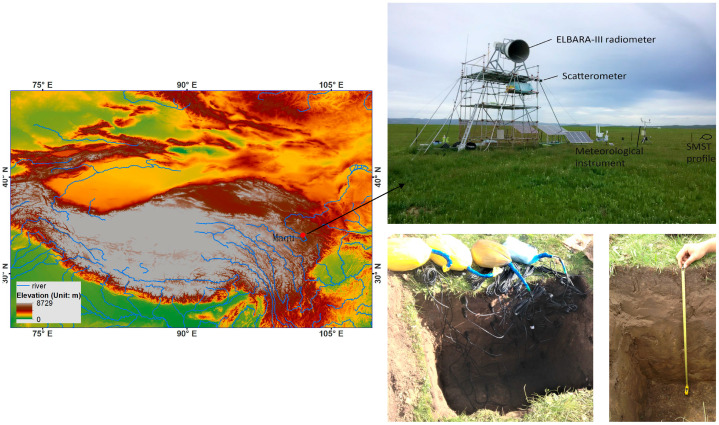
Location of the Maqu site on the northeastern Tibetan Plateau and the deployed measurements. The area of the ELBARA-III radiometer region is 25 m × 45 m [38].

The Maqu regional soil moisture and temperature (SMST) monitoring network [34,66] is located in the source region of the Yellow River on the northeastern part of the Tibetan Plateau at an altitude between 3200 m and 4200 m above mean sea level (Figure 1). The Maqu area has a cold climate with dry winter and warm summer (Dwb) in the updated Köppen–Geiger climate classification [67]. The winter season is from late November to late March, in which soils undergo freeze–thaw cycles [38]. Land cover is mainly alpine meadows, with grass heights varying from 5 to 15 cm throughout the growing season due to intensive grazing by livestock (e.g., yaks and sheep). The prevailing soil types are sandy loam, silt loam and organic soil with an average of 30.3% sand and 9.9% clay and a maximum of 39.0% organic matter [23]. The soil profile at shallow depths (until 40 cm) in this region typically consists of two horizons (i.e., layers) (Figure 1). The top A mineral horizon is enriched with organic matter and other decomposed materials, and clay and easily dissolved compounds in this layer tend to leach out over time. The mineral B horizon (~20–40 cm) beneath the surface contains less organic matter and concentrated sand and silt particles [23,68]. Besides the soil property data, as reported in [38], available in situ measurements on the Maqu site (33.91° N, 102.16° E) also involve meteorological data, profile SMST, turbulent heat fluxes by the eddy-covariance system and L-band $T_{B}^{P}$ by an ELBARA-III microwave radiometer [69] (Figure 1).

### 2.2. DA System

#### 2.2.1. Community Land Model

The Community Land Model (CLM) [60] is the land surface component of the Community Earth System Model (CESM) and can be realized offline with available atmospheric forcing. The CLM employs the Monin–Obukhov similarity theory to derive land surface fluxes and a modified Richards equation to predict the one-dimensional, multi-layer, vertical soil water flow, in which the Clapp and Hornberger [70] power function is used to describe soil water retention and soil hydraulic properties (i.e., SHPs). Therein, the four hydraulic parameters—saturated SM content $\theta_{sat}$ (m^3^/m^3^), saturated matric potential $\psi_{sat}$ (mm), pore size distribution index B (dimensionless) and saturated hydraulic conductivity $k_{sat}$ (mm/s)—are estimated through the Cosby et al. [71] PTFs with the inputting of percentages of sand and clay and the organic matter fraction [72]. In CLM v4.5, soils are divided into 15 layers, where the soil layer node depth defines where the volumetric soil water and soil temperature are estimated. The depths of the soil layers exhibit an exponential relationship along the profile in the CLM (see Equation (6.5) in [60]). Table 1 lists the depth information for the ten layers of the soil column used in SM estimations. The De Vries [73] thermal parameterization scheme, instead of the default Johansen [74] scheme, is adopted in this study to estimate soil thermal properties (i.e., soil heat capacity and thermal conductivity), given its physical consideration and higher performance based on the in situ investigations [23,75]. Other model physics and parametrizations are applied in default schemes in this study, in which the vegetation above the soil surface is characterized by the defined plant functional types (PFTs).

In this paper, the collected half-hourly atmospheric data involving wind speed, near-surface air temperature, near-surface relative humidity and air pressure, liquid precipitation, and incident solar and longwave radiation on the Maqu site [38] is taken as the driving force in the CLM realization. Time series Leaf Area Index (LAI) data extracted from the MCD15A2H-MODIS/Terra + Aqua LAI is used to define the corresponding PFT.

#### 2.2.2. Tor Vergata (TVG) Model

The TVG model [58,76] assumes that the soil acts as a homogeneous infinite half-space with a rough interface, and the overlying vegetation is represented as an ensemble of discrete dielectric scatters. The scattering modeled by the TVG model involves three components: vegetation volume scattering, soil surface scattering and the interaction between vegetation and soil.

In TVG, the grass leaves on the Maqu site are described as dielectric thin discs, which exhibits a random orientation distribution [53]. Bistatic scattering and extinction (absorption plus scattering) cross-sections of the scatter are computed by the Rayleigh–Gans approximation at the L-band [77], in which the Matzler [78] model is used to calculate the vegetation dielectric constant. Subsequently, the contributions of all vegetation scatters (discs) are integrated by using the matrix doubling algorithm, and the scattering and transmission matrices of the whole vegetation are then obtained. Values of the vegetation parameters, such as the grass leaf radius and thickness and leaf moisture content used in this study, are calibrated values from [53] and [79], which are found to be insensitive to the emissivity in the L-band [80]. The aforementioned LAI product in Section 2.2.2 is also used to determine the number of leaves (i.e., equals LAI/leaf area).

Soil surface scattering is computed by the AIEM [57] with the inputting of the soil dielectric constant and the surface roughness parameters (i.e., the standard deviation of surface heights s of 0.9 cm, correlation length of surface height L of 9 cm and the exponential autocorrelation function in this case [59]), which are the satellite observation calibrated results [53]). In this study, the effective dielectric constant of the air-to-soil medium is derived based on the developed ATS model [59], in which the dielectric constant of bulk soil that acts as the lower boundary of the ATS zone is calculated with a soil dielectric mixing model developed by Park et al. [45,81]. The Park model considers the effect of organic matter (by linking its content to estimate the dry bulk density and, therefore, soil porosity) on the soil dielectric constant. Its inputs involve volumetric SM, soil temperature, sand and clay fractions and organic matter content. The formulations of the Park model are listed in the Supplementary Materials.

With the above computed scattering for vegetation and soil parts, respectively, the same matrix doubling algorithm is used to combine these two and obtain their interaction contributions. The scattering coefficients in the backward direction $\sigma^{0}$ are then directly obtained. By integrating the bistatic scattering coefficients over the half-space above the surface and applying the energy conservation law, the emissivity $e_{p}\left(\theta_{i}\right)$ under p polarization (i.e., H or V) at an incidence angle $\theta_{i}$ is obtained. Given the low vegetation emission in the L-band [39,82,83], the physical temperature of vegetation is assumed to be the same as that of soil. The effective soil temperature $T_{eff}$ is estimated with the Wilheit [84] coherent model, which considers the impact of the SM and temperature profile on soil microwave emission. Finally, $T_{B}^{p}$ (= $e_{p}\left(\theta_{i}\right)\cdot T_{eff}$) is computed by the emissivity $e_{p}\left(\theta_{i}\right)$ multiplying $T_{eff}$. For the detailed flowchart of the forward $T_{B}^{p}$ simulation by this integrated AST-AIEM-TVG model, refer to Figure 2 in [59].

#### 2.2.3. The Local Ensemble Transform Kalman Filter (LETKF)

The DasPy data assimilation system [61,62] was developed to integrate observations from multiple sources with the CLM to improve predictions of the water, carbon and energy cycles of the soil–vegetation–atmosphere continuum. The incorporated LETKF [65] DA algorithm uses the Gaussian approximation and follows the time evolution of the mean and covariance (also called uncertainties in DA) by propagating an ensemble of states. The derivation of the LETKF given in [65] is summed up in the Supplementary Materials. To ensure a stable ensemble Kalman filter operation, localization and inflation are required to address sampling errors, as described in [85,86]. In DasPy, the multiplicative inflation algorithm, as defined in [87], is applied to eliminate residual sampling errors and spread the ensemble of soil properties and SM. As there is only one observation site in this study, the spatial localization implemented in the LETKF for reducing spurious spatial error correlations is not used, while the time localization is implemented, as the assimilation is conducted during a specific period.

#### 2.2.4. $T_{B}^{p}$ Observations

The SMAP Level-1C $T_{B}^{p}$ data acquired by the L-band radiometer at 2–3-day intervals [88] were extracted on the Maqu point site (33.91° N, 102.16° E) [38]. SMAP $T_{B}^{p}$ at near 6:00 AM local time (in descending pass) is used for assimilation, because the temperature within the vegetation–soil continuum can be assumed to be homogeneous at this time. The vegetation temperature is thus set the same as the soil temperature in the forward $T_{B}^{p}$ simulations in this study. The different spatial scales of SMAP $T_{B}^{p}$ (with spatial resolution of 9 km) and observations on the field (with meter scale) have not been taken into account in this study. SMAP $T_{B}^{p}$ observations used in the study are the arithmetic average of the fore- and aft-looking data. Furthermore, the ELBARA-III measured $T_{B}^{p}$ at the field scale is used for evaluation. The detailed descriptions of the instrumentation and data on the Maqu site are given in [38].

### 2.3. Experimental Design

In this study, the model physics, structure and parameterization involving the formulation of the PTFs described in Section 2.2.1 are assumed to be ideal. Therefore, the uncertainties that affect the model prediction performance (formulated as variance and covariance in DA) mainly come from the basic soil properties and meteorological forcing data. In practice, the uncertainties in the two types of data are unavoidable, as there are always uncertainties in the measurements (https://www.bipm.org/en/committees/jc/jcgm/publications) (accessed on 23 February 2023). As described in Section 1, the errors in the soil property datasets are further exaggerated by limited soil profiles and uncertainties in covariates and interpolation accuracy. To carry out investigations on whether SMAP $T_{B}^{p}$ assimilation improves estimates of soil properties, four experiments, including a reference, an open loop and two types of data assimilation strategies, are designed for evaluations and comparisons. Therein, the SoilGrids1km dataset [89] is selected, as it is reported to yield better accuracy over other global and regional datasets on the Tibetan Plateau [23]. The SoilGrids1km dataset is then used to prescribe property data for the CLM ten soil layers (see Table 1) through linear interpolation. The experiments are conducted during the soil-thawed period in this study, considering that (1) the soil freeze–thaw processes during the winter period (from November afterward, in this case) might reconstruct topsoil particle composition and alter soil physical properties [90,91]; (2) snowfall and soil freeze–thaw complicate the microwave emission and render the $T_{B}^{p}$ modeling not reliable [92]; (3) the soil hydraulic properties can be substantially impacted and altered by the presence of ice in the soils [75,93,94].

The reference (unperturbed, single-ensemble and denoted as Ref) run is driven by the in situ atmospheric forcing data over the period from 1 May 2016 to 30 October 2016 after the spin up to produce SMST profiles and land surface fluxes. The results of the Ref experiment are used to evaluate the accuracy of the DA experiments.

To conduct the open loop and data assimilation experiments, both atmospheric forcing data and basic soil property data are perturbed, as uncertainties are assumed from these two sources, as previously mentioned. Specifically, the in situ atmospheric forcing data of precipitation, air temperature and radiation are perturbed, given their dominance in influencing SM, soil temperature and associated $T_{B}^{p}$. The corresponding perturbation parameters and their values according to [95] are listed in Table 2. The SoilGrids1km data on the Maqu site are perturbed by adding small, uniformly distributed noise, as shown in Table 3, given its aforementioned good accuracy. Specifically, the geoR package (https://rdrr.io/cran/geoR/man/grf.html, last accessed on 26 February 2023) of statistical data analysis software R is used to generate a Gaussian random noise field. The perturbed value is then the sum of the original values extracted from the SoilGrids1km dataset and the defined Gaussian noise field.

Given the fact that the near-surface (~2.5 cm) soil contributes the most to soil emission in the L-band [84,96], the soil properties of the first layer in the CLM are perturbed and retrieved in this study. Soil properties at the other depths are obtained by using a prior depth ratio, considering the typical development of soil and its profiles (i.e., pedogenesis [68]). As described in Section 2.1, the organic matter content decreases and the sand fraction increases along the depth. Utilizing the measurements of soil property profiles [23] and the exponential formulation adopted by the CLM (Equation (6.5) in [60] to obtain fine soil layers near the soil surface), the obtained depth ratios are obtained for the ten soil layers regarding the organic matter content and sand fraction at the Maqu site (see Table 1). The clay fraction experiences very small changes (within 2%) with the depth [23]. Therefore, the prior depth ratio for the clay fraction is set to 1.

Eventually, the 30 ensemble members with these perturbed atmospheric forcing data and soil parameters are obtained. An open loop (denoted as OL) experiment then is realized without implementing DA. It is noted that the selected ensemble size of 30 considers the effectiveness in balancing the ensemble performance and the cost of computational resources, as reported by [61,97].

**Figure 2:**
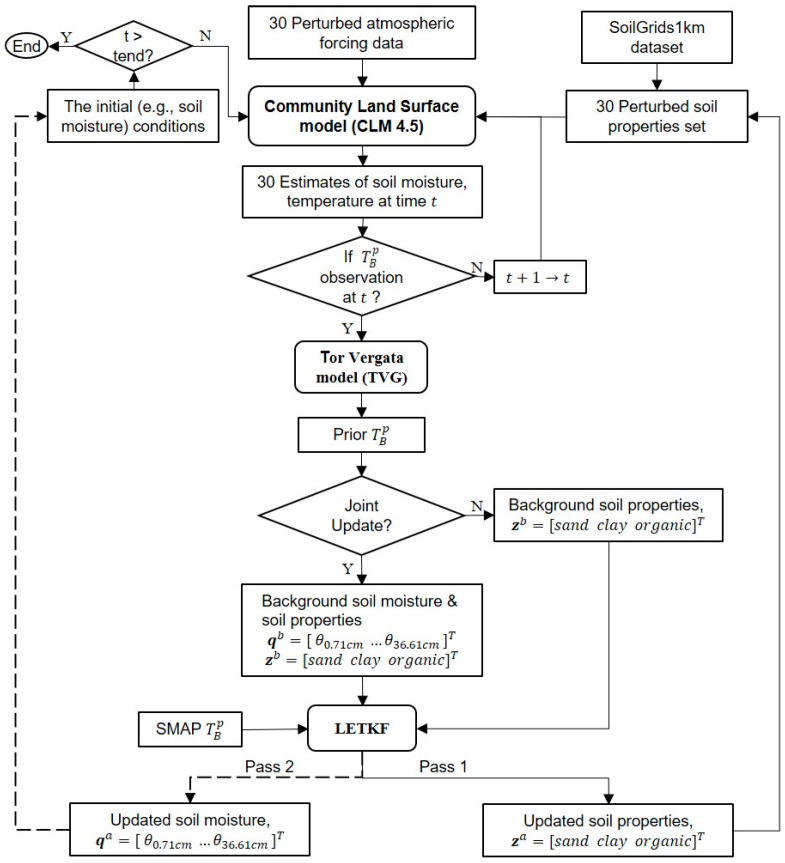
Flowchart of the retrieval of basic soil physical properties and the update of soil moisture in the DasPy data assimilation system. The rounded rectangles indicate the following four parts: the system model CLM, observation operator TVG model, LETKF data assimilation algorithm and SMAP $T_{B}^{p}$ observations. The black arrows denote forward flows in soil property retrieval and the dash arrows denote soil moisture update. t denotes time, t+1 is the next time step, and the ensemble size is 30. (Note: in the only_Para experiment, only Pass1 is used; in the Joint_Updt experiment, both Pass1 and Pass2 are used to update values of the soil properties and SM, respectively.)

The third and fourth experiments retrieve soil properties using the DA strategies over the soil-thawed period from 1 May 2016 to 31 August 2016. To identify whether the retrieved soil properties alone are sufficient to help improve estimates of land state variables (i.e., SMST) and land surface fluxes (i.e., sensible heat and latent heat) by the CLM, the third experiment updates only soil properties, i.e., without updating SM (denoted as Only_Para). The fourth experiment (denoted as Joint_Updt) updates both the soil properties and soil moisture during the assimilation period, based on the state augmentation method [98] and a dual-pass approach implemented in Daspy (Figure 2) for saving computation memory.

In the two DA experiments, the ensemble means of soil properties, rather than the perturbed property values for each member, are fed into the TVG model, as they are reported being more appropriate in parameter retrieval [61]. Moreover, the SMST of the second layer (2.79 cm in Table 1) is prescribed as those of the first layer in the TVG model, because, from the theoretical point of view, the penetration depth of SM is about 1/10 of the wavelength of observation (~2.5 cm for the L-band) [84,99]. The simulated SMST of the following four layers (i.e., until 36.61 cm) are also fed into the TVG model for effective soil temperature calculations with the Wilheit model (see Section 2.2.2), since the in situ measured soil moisture at 40 cm remains almost unchanged during the study periods [59]. As such, the SM of only the first six layers is updated in the Joint_Updt experiment. Additionally, in each assimilation step, the values of the perturbed soil properties (i.e., the sum of the sand fraction and clay fraction and the range of organic matter density) are checked and modulated based on the values listed in Table 2. The ranges of retrieved values of soil properties are checked, as well. Figure 2 shows the flowchart of the DA experiments conducted in this study.

Finally, the retrieved soil properties obtained at the final assimilation step are compared to the in situ measurements with the root mean square errors (RMSEs) calculated. Therein, the clay fraction retrieved at high accuracy, to a great degree, is assumed in this study to reflect the success of soil property retrieval, given its outstanding roles in estimating SM and soil dielectric constant. That is, clay particles with platy-like structures contribute more to the overall specific surface of the soil for holding moisture than sand and silt particles with a smooth surface do [15]. Moreover, the clay surface, which carries a net negative electrostatic charge, retains the adsorbed water. The adsorbed water is also called bound water in the soil dielectric model and reported to exhibit a different dielectric constant from that of free soil water [81,100,101]. Furthermore, the sensitivity analyses (not shown) also reveal that the variation of the clay fraction results in estimated SM values changing greater than those due to the variations of the sand and organic matter fractions. Last but not least, the four experiment results are compared to the in situ observations for evaluating the performance of SMAP $T_{B}^{p}$ assimilation in estimating SMST and land surface fluxes, as well as supporting the discussion of uncertainties (related to the soil physical properties) in the model physics and structure.

## 3. Results and Discussions

### 3.1. Retrieved Soil Physical Properties

**Figure 3:**
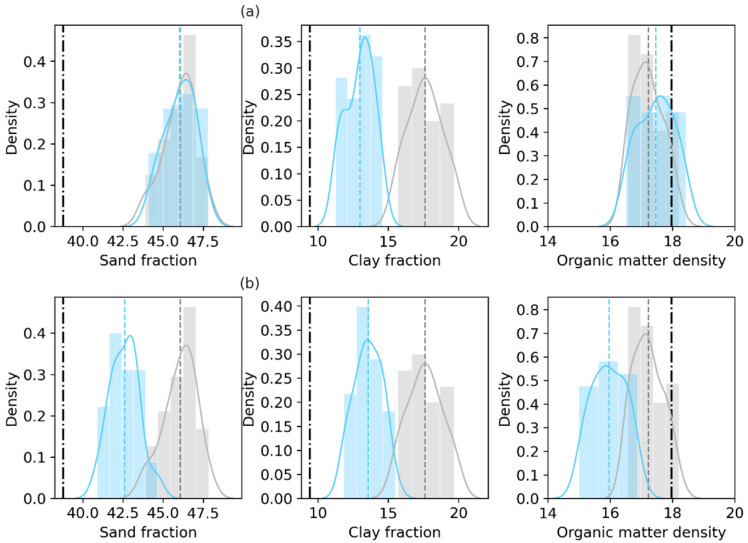
Prior and posterior distributions of the sand fraction (%), clay fraction (%) and organic matter density (kg/m^3^) of the first layer, with the truth based on laboratory measurements of the 0–5 cm soil layer sampled in the field. The top panel labeled (**a**) displays the Only_Para experiment results based on SMAP $T_{B}^{H}$ assimilation. The bottom panel (**b**) displays the Joint_Updt experiment results. In each subfigure, gray indicates the prior, light blue indicates the posterior, and the black dash-dotted line indicates the laboratory measurements.

Figure 3 shows the prior (gray) and posterior (blue) distributions of the retrieved sand fraction (%), clay fraction (%) and organic matter density (kg/m^3^) of the first layer obtained at the final assimilation step in the Only_Para and Joint_Updt experiments by assimilating SMAP $T_{B}^{H}$, with the truth based on laboratory measurements of the 0–5 cm soil layer sampled in the field. The Only_Para experiment results show that the retrieved sand fraction exhibits almost the same distribution as that of the prior (the overlapped gray and light blue lines in Figure 3a), and both of their mean values are larger than the truth (the solid black vertical line in Figure 3) within 8% (Table 4). The posterior distribution of the retrieved organic matter content exhibits a slight shift toward the truth (Figure 3a). The posterior distribution of the retrieved clay fraction is obviously shifted and narrowed (a standard deviation of 1.21 vs. 0.97 in Table 4) to approach the truth (Figure 3a).

The Joint_Updt experiment results show that the posterior distributions of both the retrieved sand fraction and clay fraction shift toward the truth (Figure 3b). The improvement of sand fraction retrieval is observed in the Joint_Updt experiment, but not in the Only_Para experiment (as shown in Figure 3a). The Joint_Updt assimilation is observed to impose a negative impact on the retrieval of organic matter density, as the distribution of the retrieved organic matter density deviates from the prior and the truth (shown in Figure 3b and a standard deviation of 0.47 vs. 0.54 in Table 3). Table 5 shows that the Joint_Updt experiment reduces the RMSE by 46.6% for the sand fraction over by 0.4% with the Only_Para experiment. The Only_Para experiment reduces the RMSE for the organic matter density (14.6%), while the Joint_Updt experiment degrades the RMSE (2.08 vs. 0.89), which indicates its high negative efficiency in organic matter density retrieval. Nevertheless, both data assimilation experiments lead to the largest reduction in RMSE for the clay fraction (>48%).

**Figure 4:**
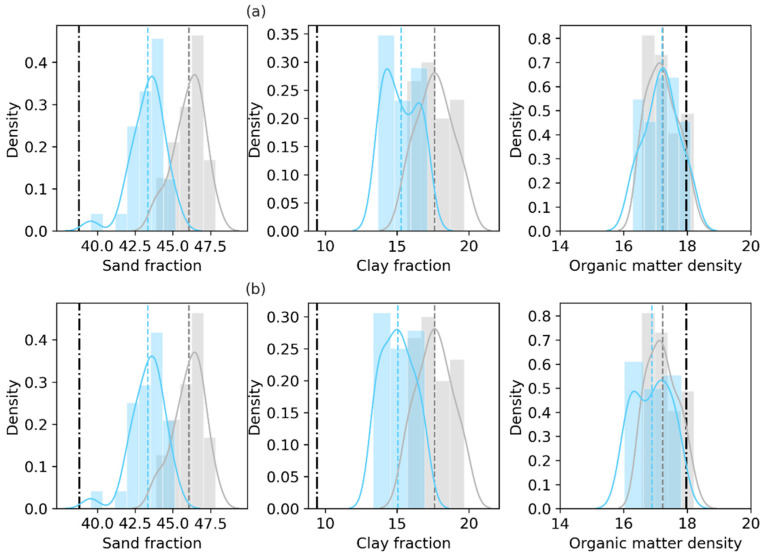
Same as Figure 3, but based on the experiments with SMAP $T_{B}^{V}$ assimilation. The top panel labeled (**a**) displays the Only_Para experiment results based on SMAP $T_{B}^{H}$ assimilation. The bottom panel (**b**) displays the Joint_Updt experiment results. In each subfigure, gray indicates the prior, light blue indicates the posterior, and the black dash-dotted line indicates the laboratory measurements.

The two DA experiments with SMAP $T_{B}^{V}$ assimilation results show that the posterior distributions of both the retrieved sand fraction and the clay fraction shift toward the truth (Figure 4). Although neither experiment achieves a positive efficiency regarding the retrieval of organic matter density (negative values in Table 5), both DA experiments do reduce the RMSEs by ~36% for the sand fraction and by ~28% for the clay fraction.

**Figure 5:**
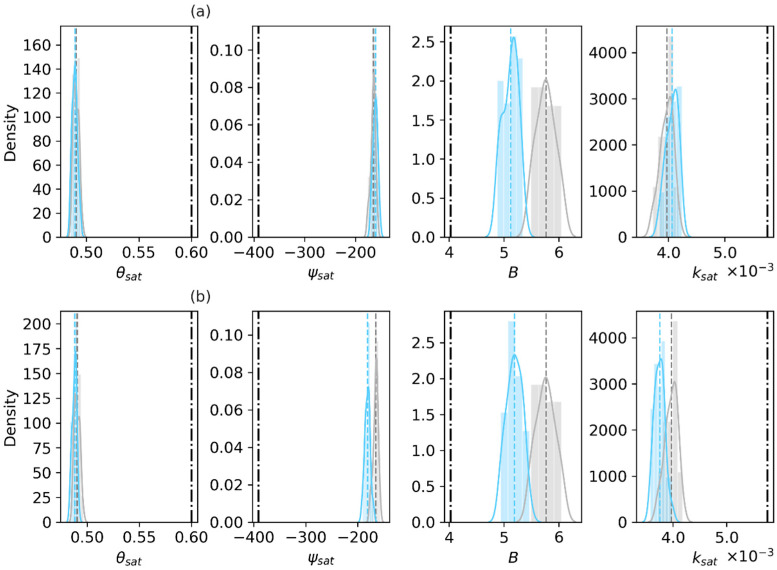
Prior and posterior distributions of the soil hydraulic parameters at 2.79 cm, with the truth based on laboratory measurements of the 0–5 cm soil layer sampled in the field. Gray shows the prior, light blue indicates the posterior, and the black dash-dotted line indicates the laboratory measurements. (**a**) Only_Para and (**b**) Joint_Updt experiments with SMAP $T_{B}^{H}$ assimilation. The hydraulic parameters are the saturated soil moisture content $\theta_{sat}$ (m^3^/m^3^), saturated matric potential $\psi_{sat}$ (mm), the pore size distribution index B (dimensionless) and saturated hydraulic conductivity $k_{sat}$ (mm/s).

**Figure 6:**
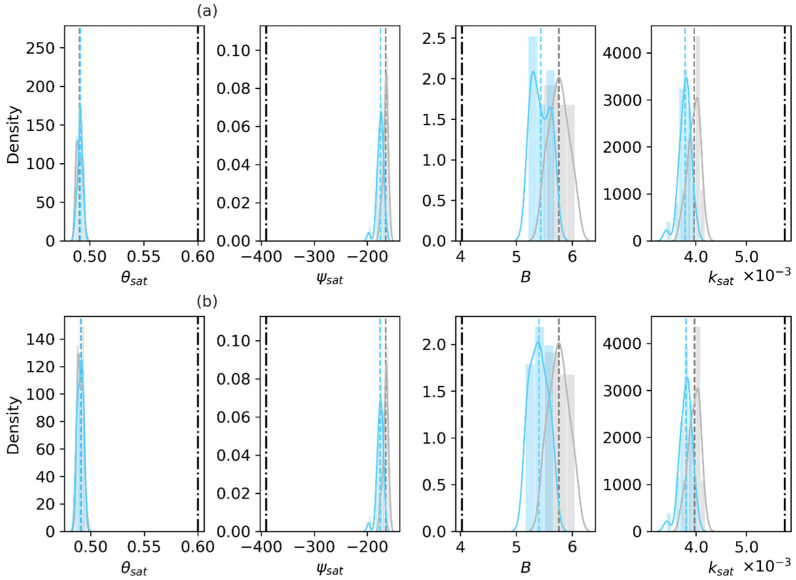
Same as Figure 5, but based on the experiments with SMAP $T_{B}^{V}$ assimilation. (**a**) Only_Para and (**b**) Joint_Updt experiments with SMAP $T_{B}^{H}$ assimilation. The hydraulic parameters are the saturated soil moisture content $\theta_{sat}$ (m^3^/m^3^), saturated matric potential $\psi_{sat}$ (mm), the pore size distribution index B (dimensionless) and saturated hydraulic conductivity $k_{sat}$ (mm/s).

Based on the retrieved basic soil properties, the four soil hydraulic parameters are obtained through the Cosby PTFs. Figure 5 and Figure 6 show that the prior and posterior values of the hydraulic parameters deviate from the in situ measurements. Both the estimated $\theta_{sat}$ and $k_{sat}$ values at 2.79 cm are lower than the measurements, and both $\psi_{sat}$ and B are overestimated. When only the soil properties are updated in the Only_Para experiment (Figure 5a), B decreases due to the reduced retrieved clay fraction, and the other three parameters retain the same values where the posteriors of the sand fraction and organic matter density, with no appreciable differences from the prior (shown in Figure 3a). This indicates that the change in clay fraction mainly affects B. In contrast, the Joint_Updt experiment assimilating $T_{B}^{H}$ (shown in Figure 3b) and both experiments assimilating $T_{B}^{V}$ (shown in Figure 4) estimate reduced posterior sand fractions, resulting in the decrease of $k_{sat}$ and $\psi_{sat}$ (Figure 5b and Figure 6).

In short, in the Only_Para experiment, the use of $T_{B}^{H}$ is found to be more sensitive to retrievals of the clay fraction and organic matter; comparatively, the use of $T_{B}^{V}$ assimilation shows the sensitivity to the retrieval of sand fraction. This may be related to the plate-like structure of clay. In contrast, the Joint_Updt experiment with either $T_{B}^{H}$ or $T_{B}^{V}$ assimilation can retrieve both the sand and clay fractions. Nevertheless, all reductions in the RMSE values for the clay fraction indicate the improvement of soil property estimates by assimilating SMAP $T_{B}^{p}$.

Furthermore, utilizing the prior depth ratio (see Section 2.2.4), the posterior distributions of the retrieved clay fraction and sand fraction of the third layer (i.e., 11.89 cm) (as shown in Figure S1 and Tables S1 and S2 in the Supplementary Materials, as an example) also shift toward the truth. This indicates that by updating the soil properties of the first layer, the descriptions of the soil properties at the other depths can be enhanced through the prior depth ratio. However, due to the fixed PTF structures, the derived soil hydraulic parameters exhibit slight changes.

### 3.2. Estimates of SM

**Figure 7:**
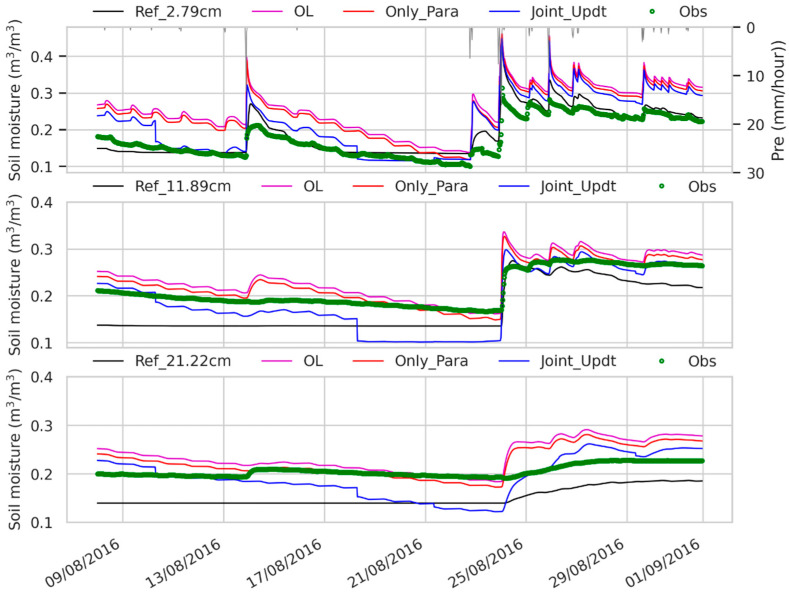
Soil moisture time series from 7 August 2016 to 31 August 2016 of the second (2.79 cm), third (11.89 cm) and fourth (21.22 cm) layers in the reference (Ref), open loop (OL) experiments, experiment with only soil properties updated (Only_Para) and experiment with both soil properties and soil moisture estimates (Joint_Updt) by assimilating SMAP $T_{B}^{H}$. Obs denotes profile soil moisture measured by 5TM ECH2O probes deployed at the ELBARA-III radiometer region on the Maqu site [38].

**Figure 8:**
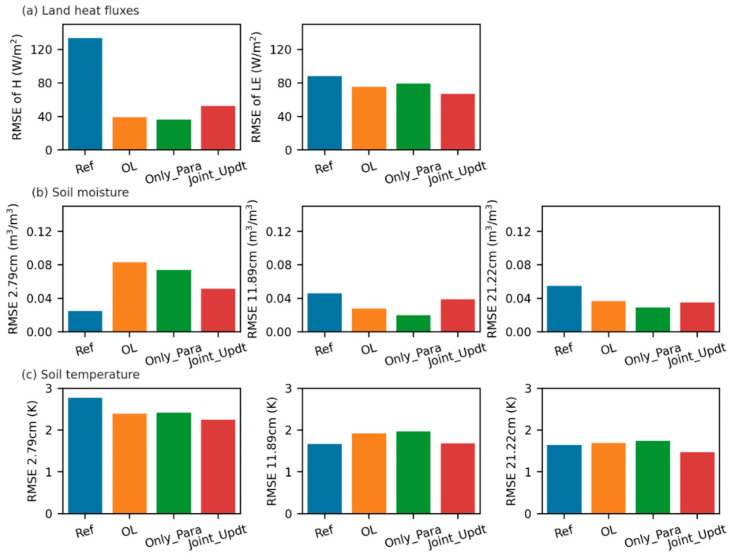
RMSE values of the land state and flux variables for the reference (Ref) experiment, open loop (OL) experiment, experiment with only soil properties updated (Only_Para) and experiment with both soil properties and soil moisture estimates (Joint_Updt) by assimilating SMAP $T_{B}^{H}$ over the assimilation period (from 8 August 2016 to 31 August 2016), in comparison to the in situ observations (i.e., profile soil moisture and temperature measurements and the turbulent heat fluxes measurements by eddy-covariance system deployed at the Maqu site [38]). (**a**) is for land latent heat flux (LE) and sensible heat flux (H), and (**b**) and (**c**) are for soil moisture and soil temperature at different depths, respectively.

As $T_{B}^{H}$ assimilation shows more improvements than $T_{B}^{V}$ assimilation in the clay fraction retrieval (see Section 3.1), the evaluation analysis of the soil states (SMSTs) and land latent and heat fluxes (LE and H) in the DA experiments is only presented for those with $T_{B}^{H}$ assimilation. Figure 7 shows the SM at 2.79 cm simulated in the reference experiment is close to the in situ observations (RMSE < 0.04 m^3^/m^3^ in Figure 8b), despite overestimations when the soil is wet (e.g., 25–31 August 2016). The SM at 2.79 cm simulated in the OL experiment shown in Figure 7 is much higher than the observations and yields a large RMSE (approximately 0.08 m^3^/m^3^ in Figure 8b).

After DA, the SM at 2.79 cm estimated in the Only_Para experiment is still higher than the in situ measurements, but exhibits slight improvements over the OL experiment (Figure 8, RMSE of 0.07 m^3^/m^3^ vs. 0.08 m^3^/m^3^ in Figure 8b). This suggests that the soil property with fine accuracy is not a sensitive factor affecting SM estimates. The SM at 2.79 cm estimated in the Joint_Updt experiment is closer to the observations than that estimated in the OL and Only_Para experiments (RMSE of 0.05 m^3^/m^3^ vs. 0.08 m^3^/m^3^ vs. 0.07 m^3^/m^3^ in Figure 8b), especially when the soil undergoes the drying process (e.g., approximately 19 August 2016), but SM is overestimated in the wet soil (Figure 7). This signifies that the model structure relating to surface soil moisture estimates may contain uncertainties. For instance, the Campbell [102] function used in the CLM is reported, not considering the transition zone near saturation for natural fine-textured soils [70], which results in moisture overestimation when the soil approaches saturation status.

The SM at greater depths (i.e., 11.89 and 21.22 cm) simulated in the reference experiment presents underestimations (shown in Figure 7, with a RMSE of 0.05 m^3^/m^3^ in Figure 8b) over the observations. While the SM at depths of 11.89 and 21.22 cm simulated in the OL experiment is close to the observations, despite the slight overestimations (shown in Figure 7), it yields a small RMSE (<0.04 m^3^/m^3^ in Figure 8b). The SM at depths of 11.89 and 21.22 cm estimated in the Only_Para experiment is close to that estimated in the OL run (Figure 7). This is expected because the updated soil properties of the first layer undergo only small changes over the prior soil properties (shown in Figure 3), and this results in slight changes in the updated soil properties of the deeper layers through the prior depth ratio. Combined with the fixed PTF structure, the estimated SM does not differ from that estimated by the OL experiment. However, in the Joint_Updt experiment, the SM at depths of 11.89 and 21.22 cm is jointly updated by using the calculated surface increment through the LETKF algorithm. The updated SM values are close to those obtained by the OL and Only_Para experiments, except when the surface soil becomes dry (e.g., 18 August 2016 to 24 August 2016 in Figure 7), but yield consistencies with the observations when the soil occurs under wet conditions (e.g., 25 August 2016 to 31 August 2016 in Figure 7).

To a certain degree, this can reflect the improvement in surface information propagating downward to deeper layers through assimilation. However, the improvement may be impeded by deficiencies in the modeled subsurface physical process, as the SM at greater depths (i.e., 11.89 and 21.22 cm) is found to converge to 0.1 m^3^/m^3^ during the dry period (see Figure 7). For instance, the water potential in the root collar that drives the water flux from a given soil layer is reported not considered in the CLM v4.5 used in this case and characterized through a new plant water stress parameterization based on the hydraulic theory in the released CLM v5 [103] though. Additionally, a highly discretized profile near the surface is used in the CLM, which may lead to a low coupling strength from the surface to deeper layers in the CLM, as claimed by [104], thus constraining the efficiency of the abovementioned improvements through DA. Changing the layering structure (especially near the surface) of the CLM should be tested in future research to verify if the coupling strength can be enhanced and SM, and even the basic soil properties of the deeper layers, can be updated through assimilation.

### 3.3. Estimates of Land Surface Fluxes and Soil Temperature

**Figure 9:**
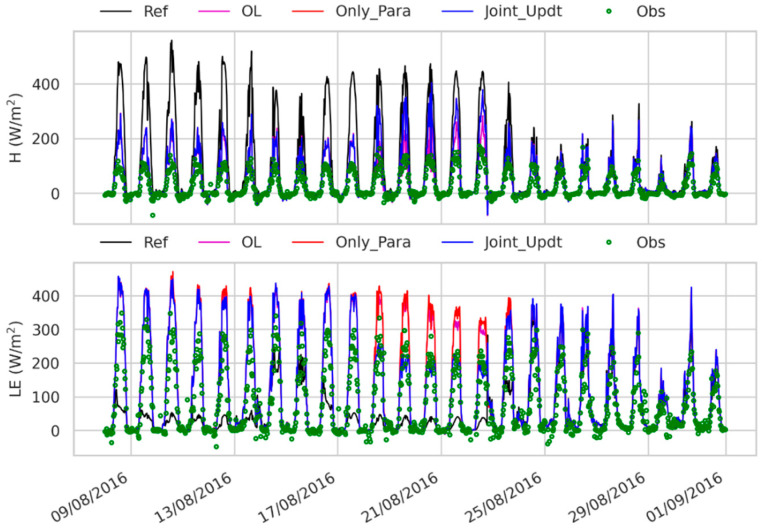
Comparison of estimated land sensible heat flux (H) and latent heat flux (LE) time series from 07 August 2016 to 31 August 2016 by the reference (Ref) experiment, open loop (OL) experiment, the scenario with only soil properties updated (Only_Para) and the scenario with both the soil properties and soil moisture estimates (Joint_Updt) updated with SMAP $T_{B}^{H}$ assimilation, to the in situ turbulent heat fluxes measurements by the eddy-covariance system deployed at the Maqu site [38].

**Figure 10:**
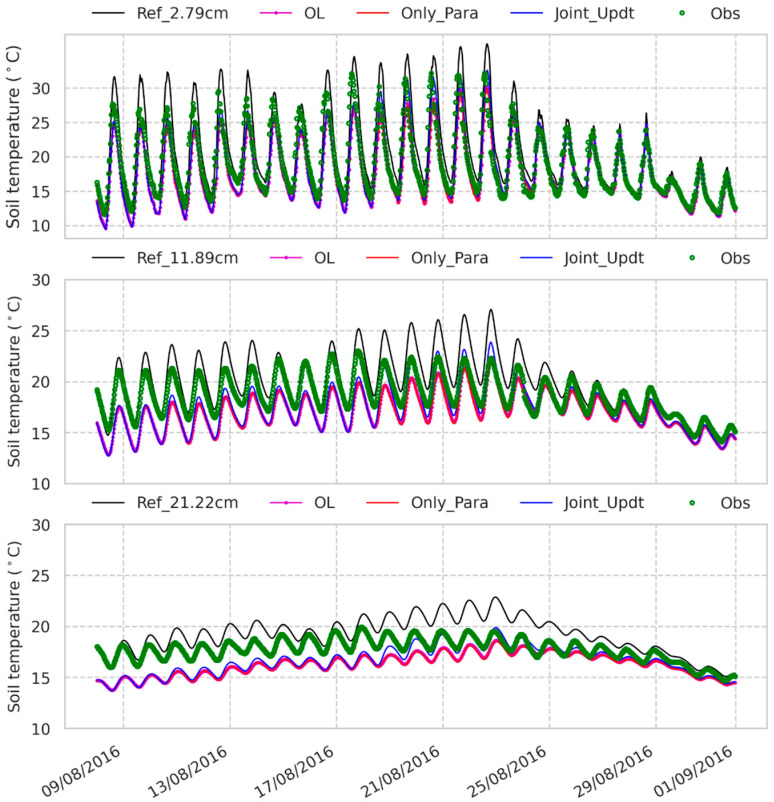
Soil temperature time series from 7 August 2016 to 31 August 2016 of the second (2.79 cm), third (11.89 cm) and fourth (21.22 cm) layers in the reference (Ref), open loop (OL) experiments, experiment with only soil properties updated (Only_Para) and experiment with both soil properties and soil moisture estimates (Joint_Updt) by assimilating SMAP $T_{B}^{H}$. Obs denotes profile soil moisture measured by 5TM ECH2O probes deployed at the ELBARA-III radiometer region on the Maqu site [38].

Figure 9 shows that the reference experiment estimates the latent heat flux (LE) lower than the observations with a large RMSE (over 80 W/m^2^ in Figure 8a). A similar large RMSE (82 W/m^2^ in this case) occurs to the reference estimated sensible heat flux (H), which is larger than the observations (Figure 9). The reference experiment produces the estimated soil temperature at the different depths with RMSE values less than 3 K when compared to the measurements (Figure 8c and Figure 10). The LE and H simulated by the OL experiment are closer to the observations (Figure 9, with smaller RMSE values in Figure 8a) than is the reference run, which might be due to the fact that the SM at the deeper layers simulated by the OL experiment is closer to the observations than that by the reference experiment. The Only_Para experiment estimates better H than the other experiments do (with smaller RMSE values in Figure 8a). In contrast, the Joint_Updt experiment simulates better LE (with smaller RMSE values in Figure 8a).

Nevertheless, these four experiments estimate H and LE with large discrepancies (RMSE over 70 W/m^2^, especially during midday time in Figure 8) compared to the measurements. The discrepancies due to soil physical properties are attributed to the ‘soil beta’ parameterization, which is employed by CLM to represent the effect of soil resistance on soil evaporation [60]. This empirical function in CLM depends on the soil moisture and field capacity of the first soil layer, and the field capacity estimate is determined by $\theta_{s}$, $K_{s}$ and B of the first soil layer. As $\theta_{s}$ and $K_{s}$ are underestimated and B is overestimated (shown in Figure 5 and Figure 6), the field capacity is underestimated. This leads to a decrease in soil resistance and, consequently, an increase in soil evaporation and lower LE simulated by the reference run during the dry period (e.g., from 9 August 2016 to 15 August 2016) than the observations, as shown in Figure 9.

### 3.4. Estimates of $T_{B}^{p}$

**Figure 11:**
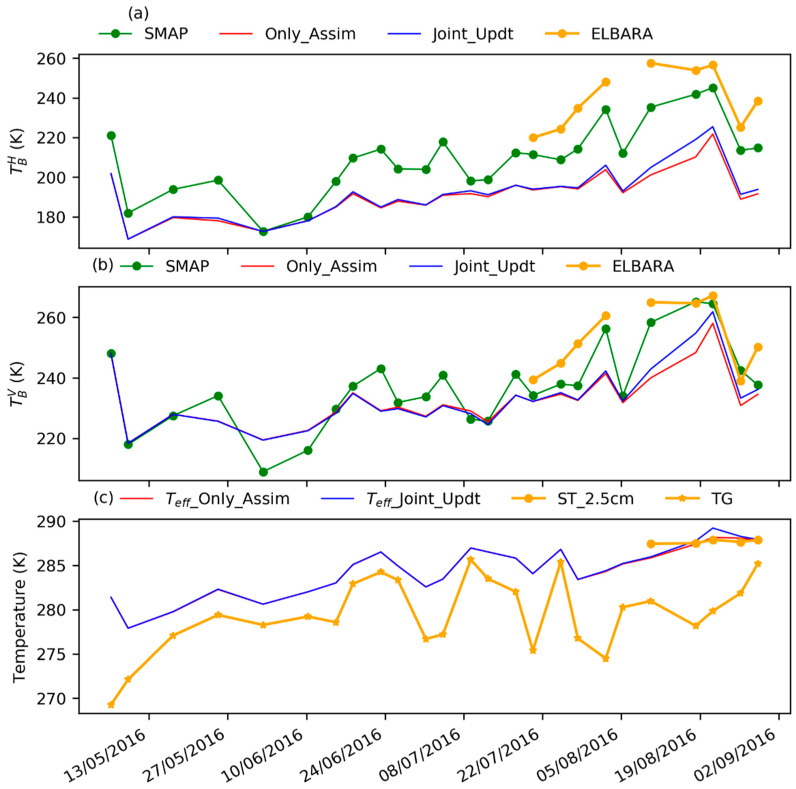
Comparisons of the SMAP $T_{B}^{p}$, $T_{B}^{p}$ estimated by the two DA experiments and the ELBARA-III observed $T_{B}^{p}$ during the assimilation period (from 1 May 2016 to 31 August 2016), as well as comparisons of $T_{eff}$. (**a**) is for $T_{B}^{H}$ comparison, (**b**) is for $T_{B}^{V}$ comparison, and (**c**) is for $T_{eff}$ comparison. The in situ soil temperature at 2.5 cm (ST_2.5cm) is available from 7 August 2016 onwards. TG denotes the ground surface temperature, which is derived based on the in situ measured downward and upward longwave radiations and the Stefan–Boltzmann equation.

We compare $T_{B}^{p}$ estimated by the two DA experiments to the SMAP and ELBARA-III observations. Figure 11a,b show that the two DA experiments underestimate $\;T_{B}^{p}$ when compared to the SMAP observations. This is mainly due to the overestimated SM (shown in Figure 7), which results in the overestimated soil dielectric constant and, thus, underestimated emissivity $e_{p}$. The estimated $T_{eff}$ shows coincidences with the in situ soil temperature at 2.5 cm from August 2016 onwards and with the observed TG during the period from May 2016 to July 2017, where the in situ soil temperature observations are not available. The difference between $T_{eff}$ and TG is confined within 5 K (Figure 11c), which is acceptable compared to the uncertainty in the estimated SM, as the latter leads to emissivity largely biased in the forward $T_{B}^{p}$ simulation. The two DA experiments in this case tend to yield lower values of SM in order to reduce the underestimation of $T_{B}^{p}$. As such, the clay fraction is preferentially reduced through assimilation, as shown in Figure 3 and Figure 4.

In contrast to $T_{B}^{H}$ assimilation, a better match is shown between the posterior $T_{B}^{V}$ values and the SMAP observations (Figure 11b). This is consistent with those reports that $T_{B}^{V}$ is less affected by SM and surface roughness changes [39,64]. The results presented in Section 3.1 indicate that $T_{B}^{H}$ assimilation is more applicable for retrieving the clay fraction and organic matter than $T_{B}^{V}$ assimilation, which may be related to the plate-like structure of clay particles and organic matter. In turn, $T_{B}^{H}$, to some degree, is sensitive to SM changes from the perspective of soil physics, which is consistent with the demonstration based on regression analysis of microwave observations [105].

Figure 11a,b also show that the SMAP $T_{B}^{p}$ at the Maqu site is consistent with the ELBARA-III observations, indicating the good quality of SMAP $\;T_{B}^{p}$ data. Due to their different spatial resolutions, the SMAP $T_{B}^{H}$ data with a spatial resolution of 36 km are lower (~15 K) than the ELBARA-III observations at the field scale (~m), similar to SMAP $T_{B}^{V}$ (lower ~5 K). The difference in vegetation dynamics between the SMAP scale (grazed) and the field scale (fenced off) may be another factor contributing to the differences in the estimated $T_{B}^{H}$ and $T_{B}^{V}$. To better estimate vegetation contribution and disentangle the soil and vegetation scattering-emission effects to further improve the performance of the observation operator and associated estimates of soil physical properties, SM and land surface fluxes with LSMs, the unified passive and active observation operator used in this study can assimilate radar backscatter products in the future, as the radar sensor products have shown great capabilities in SM retrieval and vegetation parameter estimations [106,107,108].

## 4. Conclusions

In this study, a physically-based, unified, passive and active, microwave observation operator, namely, a discrete scattering-emission model (the Tor Vergata model, or TVG model), with enhanced physical considerations is for the first time, to the best of our knowledge, coupled with CLM 4.5 in a data assimilation framework with the LETKF algorithm implemented. To investigate whether SMAP $T_{B}^{p}$ data assimilation improves estimates of soil properties and associated land states (i.e., soil moisture and temperature) and land surface fluxes, four experiments including a reference, an open loop and two types of data assimilation strategies are designed. One assimilation experiment updates only the soil properties (Only_Para), and the other updates both the soil properties and soil moisture (Joint_Updt). In situ observations at the Maqu site on the eastern Tibetan Plateau are utilized to help with the investigation. To assess the effect of the different polarization configurations on the retrieval results, SMAP $T_{B}^{H}$ and $T_{B}^{V}$ are assimilated separately.

The results show the improvement of the soil property estimates by assimilating SMAP $T_{B}^{p}$, as both assimilation experiments reduce the RMSE for the retrieved clay fractions over the in situ measurements. The descriptions of the soil properties along the profile are also improved through the retrieved soil properties of the first layer and prior depth ratio. In the Only_Para experiment, the use of $T_{B}^{H}$ is more sensitive to clay fraction and organic matter retrieval, and $T_{B}^{V}$ to sand fraction retrieval. Comparatively, the Joint_Updt experiment can retrieve both the sand and clay fractions when assimilating either $T_{B}^{H}$ or $T_{B}^{V}$. The Joint_Updt experiment also provides better estimates of the soil moisture, soil temperature and land surface fluxes during the assimilation period, except the soil drying period.

Comparing the assimilation results to the in situ observations indicates that the retrieved soil properties with a finer accuracy are not sensitive factors affecting the accuracy of the soil moisture estimates by the CLM. Uncertainties in the model structures relating to soil moisture estimates should be considered. For instance, optimizing PTF structures may be an alternative way to improve soil hydraulic property estimates and, thereby, soil moisture estimates, as more investigations [24,109] indicate consequences in soil state estimates due to uncertainties induced by PTFs structures. Moreover, the Van Genuchten [110] function, which yields good estimates of soil moisture near saturation may be used to replace the Campbell [102] function in the CLM. To enhance the surface soil moisture information propagating downward to the deeper layers through assimilation, the developed parameterization of the plant hydraulic stress within CLM 5 can be incorporated, and the highly discretized layering structure of the CLM may need to be adjusted. For better land surface fluxes simulations, the parameterizations of land surface fluxes in the CLM also need to be improved, as the estimates of the reference run exhibit large discrepancies over the in situ observations.

Last but not least, DA is appreciated as tending to estimate the state of a large dynamical system based on limited information. DA applied to geoscience exhibits complexities, because it stands on its interdisciplinary nature across the observations of systems, dynamical systems, statistics and numerical optimization [86]. Meanwhile, the DA strategy has shown its great potential of obtaining basic soil properties and furthering the land surface states and fluxes consistent in both physics and scales [25,26,27], as mentioned. The observation operator employed in this study is a unified passive and active simulator that can simultaneously model passive microwave emissions ($T_{B}^{p}$) and active backscattering coefficients. As such, the developed system is also capable of assimilating satellite radar observations, which exhibit much higher spatial resolution (e.g., meter) compared to that (e.g., km) of microwave $T_{B}^{p}\;$ observations. This is the direction for future efforts, but beyond the scope of this study.

## Figures and Tables

**Table 1:** Soil layer node depth, thickness and depth at the layer interface for the ten layers in the CLM.

Layer	Layer Node Depth (cm)	Thickness (cm)	Depth (cm)	Depth Ratio [-]	
				Organic Matter Density Sand Fraction	
1	0.71	1.75	1.75	1.0	1.0
2	2.79	2.76	4.51	0.98	1.02
3	6.23	6.23	9.06	0.95	1.06
4	11.89	11.89	16.55	0.45	1.12
5	21.22	21.22	28.91	0.28	1.14
6	36.61	36.61	49.29	0.18	1.16
7	61.98	61.98	82.89	0.12	1.18
8	103.8	103.8	138.28	0.07	1.19
9	172.76	172.76	229.61	0	1.21
10	286.46	286.46	380.19	0	1.23

**Table 2:** Perturbations in the atmospheric forcing data used in this study.

Variables	Noise	Distribution	Mean	Standard Deviation
Air temperature	additive	normal	0	1.0 K
Precipitation	multiplicative	lognormal	1.0	0.5
Shortwave radiation	multiplicative	normal	1.0	0.3
Longwave radiation	additive	normal	0	36.0 W/m^2^

**Table 3:** Perturbations in the soil property data used in this study.

Soil Properties	Noise	Distribution	Lower Value	Upper Value	Range
Sand fraction (%)	Additive	Uniform	−2.0	2.0	14–60
Clay fraction (%)	Additive	Uniform	−2.0	2.0	3–20
Organic matter density (kg/m^3^)	Additive	Uniform	−1.0	1.0	1–40

**Table 4:** Data assimilation results and their calculated standard deviations (_std) for the retrieved soil properties of the first layer. $z^{b}$ and $z^{a}$ denote background and analysis, respectively.

Soil Property	True	$z^{b}$	$z^{b}$_std	$T_{B}^{H}$			$T_{B}^{V}$				
				Only_Para		Joint_Updt		Only_Para		Joint_Updt	
				$z^{a}$	$z^{a}$_std	$z^{a}$	$z^{a}$_std	$z^{a}$	$z^{a}$_std	$z^{a}$	$z^{a}$_std
Sand fraction (%)	38.8	46.1	1.0	46.0	1.0	42.6	0.9	43.3	1.1	43.3	1.1
Clay fraction (%)	9.4	17.6	1.2	13.0	1.0	13.6	1.0	15.3	1.1	15.1	1.1
Organic matter density (kg/m^3^)	18.0	17.2	0.5	17.5	0.6	16.0	0.5	17.2	0.5	16.9	0.6

**Table 5:** RMSE values of the retrieved soil properties of the first layer in the two data assimilation experiments.

Soil Property	$z^{b}$_RMSE	$T_{B}^{H}$			$T_{B}^{V}$				
		Only_Para		Joint_Updt		Only_Para		Joint_Updt	
		$z^{a}$_RMSE	Reduction	$z^{a}$_RMSE	Reduction	$z^{a}$_RMSE	Reduction	$z^{a}$_RMSE	Reduction
Sand fraction (%)	7.3	7.3	0.4%	3.9	46.6%	4.7	36.2%	4.7	36.4%
Clay fraction (%)	8.3	3.7	55.7%	4.3	48.7%	6.0	28.1%	5.7	30.7%
Organic matter density (kg/m^3^)	0.9	0.8	14.6%	2.1	−133%	0.9	−5.6%	1.2	−38.0%

## Data Availability

The in situ measurement data used in this study are from [38] and can be accessed at https://doi.org/10.6084/m9.figshare.12058038.v1 (accessed on 23 February 2023) and https://doi.org/10.6084/m9.figshare.12728444.v1 (accessed on 23 February 2023).

## Supplementary Materials

The following supporting information can be downloaded at: https://www.mdpi.com/article/10.3390/s23052620/s1, Figure S1: Prior and posterior distributions of sand fraction (%), clay fraction (%) and organic matter density (kg/m3) at the third layer (i.e., 11.89 cm), with the truth based on laboratory measurements of the 0–5 cm soil layer sampled in the field.; Table S1: Retrieved soil properties at the third layer (i.e., 11.89 cm) and their calculated standard deviations (_std) from experiment with only soil properties updated (Only_Para) and experiment with both soil properties and soil moisture estimates (Joint_Updt) by assimilating SMAP $T_{B}^{H}$; Table S2: RMSEs of the retrieved soil properties at the third layer (i.e., 11.89 cm) from experiment with only soil properties updated (Only_Para) and experiment with both soil properties and soil moisture estimates (Joint_Updt) by assimilating SMAP $T_{B}^{H}$. References [111,112] are cited in the supplementary materials.
