# Supplementary material for: Retrieving Soil Physical Properties by Assimilating SMAP Brightness Temperature Observations into the Community Land Model
Source: Sensors (Basel). 2023 Feb 27;23(5):2620. doi: 10.3390/s23052620 (PMC10007566; doi:10.3390/s23052620)
Supplement: Supplementary file 1 [file sensors-23-02620-s001.zip › sensors-2143751-supplementary.pdf]

sensors

MDPI

Supplementary Material

Retrieving Soil Physical Properties by Assimilating SMAP Brightness Temperature Observations into the Community Land Model

Hong Zhao[1], Yijian Zeng[1], Xujun Han[2], and Zhongbo Su[1,3,*]

1 Faculty of Geo-Information Science and Earth Observation (ITC), University of Twente, Hengelosestraat 99, Enschede 7514 AE, The Netherlands
2 Chongqing Engineering Research Center for Remote Sensing Big Data Application, School of Geographical Sciences, Southwest University, Chongqing 400715, China
3 Key Laboratory of Subsurface Hydrology and Ecological Effect in Arid Region of Ministry of Education, School of Water and Environment, Chang'an University, Xi'an, 710054, China
* Correspondence: Zhongbo Su (z.su@utwente.nl)

S1 Soil dielectric mixing model developed by Park et al., (2017)

Park, et al. [1] proposed a new model for the effective dielectric constant of bare soil, which considers the soil water phase as consisting of free and bound water and the bulk behavior by including a constant damping factor with the sampling depth. This model represented by equation (S1-1) is based on dielectric average mixing [2], the physically valid average method complying with the superposition rule when the calculation is examined down to the polarizability scale [1]. The formulations are below,

$$\varepsilon_{eff} = (1-P)\varepsilon_{soil} + \theta\varepsilon_{water} + (P-\theta)\varepsilon_{air} \quad \text{(S1-1)}$$

$$\varepsilon_{soil} = V_{sand}\varepsilon_{sand} + V_{silt}\varepsilon_{silt} + V_{clay}\varepsilon_{clay} \quad \text{(S1-2)}$$

$$\varepsilon_{water} = V_{bound}\varepsilon_{bound} + V_{free}\varepsilon_{free}$$

where ε_{eff} is the effective dielectric constant. ε with $soil, sand, clay, silt$ as subscriptions refer to the dielectric constant of soil and soil compositions. V with $sand, clay, silt$ as subscriptions refer to the volumetric fraction of soil compositions with $V_{sand} + V_{clay} + V_{silt} = 1$. ε_{bound} and ε_{free} are the dielectric constants of bound water and free water. V with $bound\ and\ free$ as subscriptions refer to the volumetric fraction of bound and free water with $V_{bound} + V_{free} = 1$. ε_{air} is the dielectric constant of air ($\varepsilon'_{air} = 1, \varepsilon''_{air} = 0$). θ is volumetric soil moisture and P is soil porosity. In the Park, Behrendt, LeDrew and

Wulfmeyer [1] model, soil water content below the wilting point (WP) was assumed as bound water. With more water presenting, a linear decrease of V_{bound} with total water increase from $WP < \theta < \mathrm{P}$ was assumed, namely $V_{bound} = a + b\theta$, in which $b = -1/(P - WP)$ and $a = P/(P - WP)$. Then,

$$V_{bound} = \frac{P - \theta}{P - WP} \tag{S1-3}$$

$$V_{free} = \frac{\theta - WP}{P - WP}$$

The equation (S1-1) was modified under different soil moisture conditions,

$$\begin{aligned} \varepsilon_{eff} &= (1 - P)\varepsilon_{soil} + \theta\varepsilon_{bound} + (P - \theta)\varepsilon_{air} && \theta \le WP \\ &= (1 - P)\varepsilon_{soil} + \theta\left(\frac{P - \theta}{P - WP}\varepsilon_{bound} + \frac{\theta - WP}{P - WP}\varepsilon_{free}\right) && WP < \theta \le P \quad \text{(S1-4)} \\ &\qquad + (P - \theta)\varepsilon_{air} \\ &= (1 - \theta)\varepsilon_{soil} + \theta\varepsilon_{free} && \theta > P \end{aligned}$$

To incorporate the impact of organic matter on soil dielectric constant, the adjusted wilting point and porosity in terms of the organic matter content were used in Park, et al. [3] model. The wilting point (WP) is estimated using clay volumetric fraction (V_{clay}) and the organic matter content (OM, unit: %).

$$WP = 0.02982 + 0.089V_{clay} + 0.00786OM \tag{S1-5}$$

Following this soil porosity (P) was derived using PTFs from Tóth, et al. [4],

$$\begin{aligned} P = 0.6819 - \frac{0.06480}{OC + 1} - 0.11900BD^2 - 0.02668 + 0.1489V_{clay} + 0.0803V_{silt} \quad \text{(S1-6)} \\ + \frac{0.02321}{(OC + 1)BD^2} + 0.01908BD^2 - 0.11090V_{clay} - 0.2315V_{clay}V_{silt} \\ -0.01197V_{silt}BD^2 - 0.01068V_{clay}BD^2 \end{aligned}$$

where OC is soil organic carbon and is calculated from OM divided by 1.72. Soil dry bulk density (BD) is determined as

$$BD = -0.039OM + 1.2301 \tag{S1-7}$$

The frequency-dependent dielectric constant for free and bound water can be derived by Debye's relaxation formula, substituting:

$$\varepsilon'_{free,bound} = \varepsilon_{w\infty}^{free,bound} + \frac{\varepsilon_{w0}^{free,bound} - \varepsilon_{w\infty}}{1 + \omega^2\tau_{free,bound}^2} \quad \text{(S1-8)}$$

$$\varepsilon''_{free,bound} = \frac{\omega\tau_{free,bound}(\varepsilon_{w0}^{free,bound} - \varepsilon_{w\infty})}{1 + \omega^2\tau_{free,bound}^2}$$

$$\varepsilon_{w\infty} = 4.9$$

$$\varepsilon_{w0}^{free} = (88.045 - 0.4147T + 6.295 \times 10^{-4}T^2 + 1.075 \times 10^{-5}T^3) \cdot aST$$

$$aST = 1 + 1.613 \times 10^{-3}S \times T - 3.656 \times 10^{-3}S + 3.21 \times 10^{-5}S^2 - 4.232 \times 10^{-7}S^3$$

$$\tau_{free} = (1.1109 \times 10^{-10} + 3.824 \times 10^{-12}T + 6.938 \times 10^{-14}T^2 - 5.096 \times 10^{-16}T^3)\,\frac{b_{ST}}{2\pi}$$

$$b_{ST} = 1 + 2.282 \times 10^{-5}S \times T - 7.638 \times 10^{-4}S - 7.760 \times 10^{-6}S^2 + 1.105 \times 10^{-8}S^3$$

ε_{w0} is the static dielectric constant of free water and $\varepsilon_{w\infty}$ is the minimum static dielectric constant of free water. τ_{free} is the dielectric relaxation time for free water. T and S used in these equations are soil temperature (◦C) and salinity (%). ω is the angular frequency and is calculated by $\omega = 2\pi f$ in which f is the frequency (unit: Hz). The bound water relaxation is frequency-dependent ($\omega = 2\pi f$) and soil texture-dependent relating to clay volume fraction using the equations below,

$$\tau_{bound} = 10^{-11}s \quad \text{(S1-9)}$$

$$\varepsilon_{w0}^{bound} = -36 \cdot V_{clay} + 44$$

The effective conductivity σ needs to consider for additionally accounting for the effective dielectric loss. The final soil mixture's dielectric constant ε^* is described as the real part of effective polarization ε'_{eff} and the imaginary part ε''_{eff} with effective conductivity σ_{eff},

$$\varepsilon^* = \varepsilon'_{eff} + i(\varepsilon''_{eff} + \frac{\sigma_{eff}}{w\varepsilon_0}) \quad \text{(S1-10)}$$

where ε_0 is dielectric constant for free space valued at 8.854*10-12 F.m-1. The computation of σ_{eff} is similar to that for the dielectric constant (see equation (S1-4)) as in below,

$$\sigma_{eff} = (1 - P)\sigma_{soil} + \theta(\frac{\theta}{WP}\sigma_{bound} + \tilde{\sigma}_{salt}) + (P - \theta)\sigma_{air} \qquad \theta \le WP$$

$$= (1-P)\sigma_{soil} + \theta\left(\frac{P-\theta}{P-WP}\sigma_{bound} + \frac{\theta-WP}{P-WP}\sigma_{free} + \tilde{\sigma}_{salt}\right) + (P-\theta)\sigma_{air} \qquad WP < \theta \le P \quad \text{(S1-11)}$$

$$= (1-\theta)\sigma_{soil} + \theta(\sigma_{free} + \tilde{\sigma}_{salt}) \qquad \theta > P$$

The dry effective conductivity σ_{soil} consists of the sub-phases and calculated,

$$\sigma_{soil} = V_{sand}\sigma_{sand}^{min} + V_{silt}\sigma_{silt}^{min} + V_{clay}\sigma_{clay}^{min} \quad \text{(S1-12)}$$

Conductivity for bound and free water $\sigma_{bound,free}$ are calculated,

$$\sigma_{soil} \approx \sigma_{boud} \quad \text{(S1-13)}$$

$$\sigma_{free} = V_{sand}\sigma_{sand}^{max} + V_{silt}\sigma_{silt}^{max} + V_{clay}\sigma_{clay}^{max}$$

where $\sigma_{sand}^{max} = 30 \times 10^{-3}$ and $\sigma_{sand}^{min} = 0.3 \times 10^{-3}$ S/m, $\sigma_{silt}^{max} = 75 \times 10^{-3}$ and $\sigma_{silt}^{min} = 4 \times 10^{-3}$ S/m and $\sigma_{clay}^{max} = 600 \times 10^{-3}$ and $\sigma_{clay}^{min} = 20 \times 10^{-3}$ S/m.

The geometrical structure and thickness also affect the dielectric constant of a three-dimensional medium. Assuming the soil is isotropic, the damping effect within the sampling depth can be quantified as an approximate constant of 0.8. The Park model is formulated as,

$$for\ \theta < WP \quad \text{(S1-14)}$$

$$\varepsilon'_{eff} = 0.8\big((1-P)(V_{sand}\varepsilon'_{sand} + V_{silt}\varepsilon'_{silt} + V_{clay}\varepsilon'_{clay}) + \theta\varepsilon'_{bound} + (P-\theta)\varepsilon'_{air}\big)$$

$$\varepsilon''_{eff} = 0.8((1-P)\big(V_{sand}\varepsilon''_{sand} + V_{silt}\varepsilon''_{silt} + V_{clay}\varepsilon''_{clay}\big) + \theta\varepsilon''_{bound} + (P-\theta)\varepsilon''_{air} + \frac{1}{\omega\varepsilon_0}\big(\theta(\sigma_{bound} + \sigma_{25\,°C}e^{-\varphi})\big) + (P-\theta)\sigma_{air} + (1-P)\sigma_{soil}))$$

$$for\ WP < \theta \le P \quad \text{(S1-15)}$$

$$\varepsilon'_{eff} = 0.8\left(\begin{array}{c}(1-P)\big(V_{sand}\varepsilon'_{sand} + V_{silt}\varepsilon'_{silt} + V_{clay}\varepsilon'_{clay}\big)\\ +\theta\left(\frac{P-\theta}{P-WP}\varepsilon'_{bound} + \frac{\theta-WP}{P-WP}\varepsilon'_{free}\right) + (P-\theta)\varepsilon'_{air}\end{array}\right)$$

$$\varepsilon''_{eff} = 0.8((1-P)\big(V_{sand}\varepsilon''_{sand} + V_{silt}\varepsilon''_{silt} + V_{clay}\varepsilon''_{clay}\big) + \theta\left(\frac{P-\theta}{P-WP}\varepsilon''_{bound} + \frac{\theta-WP}{P-WP}\varepsilon''_{free}\right) + (P-\theta)\varepsilon''_{air} + \frac{1}{\omega\varepsilon_0}\left(\theta\left(\frac{P-\theta}{P-WP}\sigma_{bound} + \frac{\theta-WP}{P-WP}\sigma_{free} + \sigma_{25\,°C}e^{-\varphi}\right)\right)$$

$$+(P-\theta)\sigma_{air}+(1-P)\sigma_{soil}))$$

for $P \leq \theta$: (S1-16)

$$\varepsilon'_{eff}=0.8\begin{pmatrix}(1-\theta)\left(V_{sand}\varepsilon'_{sand}+V_{silt}\varepsilon'_{silt}+V_{clay}\varepsilon'_{clay}\right)\\ +\theta\varepsilon'_{free}\end{pmatrix}$$

$$\varepsilon''_{eff}=0.8((1-\theta)\left(V_{sand}\varepsilon''_{sand}+V_{silt}\varepsilon''_{silt}+V_{clay}\varepsilon''_{clay}\right)+\theta\varepsilon''_{free}$$

$$+\frac{1}{\omega\varepsilon_0}\left(\theta\left(\sigma_{free}+\sigma_{25\,°C}e^{-\varphi}\right)\right)+(1-\theta)\sigma_{soil}))$$

Conductivity for saline water at $25\,°C$ $\sigma_{25\,°C}$ and φ are calculated,

$$\sigma_{25\,°C}(S)=0.18252S-1.4619\times 10^{-3}S^2+2.093\times 10^{-5}S^3-1.282\times 10^{-7}S^4 \quad \text{(S1-17)}$$

$$\varphi(S,25-T)=(25-T)(2.033\times 10^{-2}+1.266\times 10^{-4}(25-\mathrm{T})$$

$$+2.464\times 10^{-6}(25-\mathrm{T})^2-1.849\times 10^{-5}\mathrm{S}$$

$$+2.551\times 10^{-7}(25-\mathrm{T})S-2.551\times 10^{-8}(25-\mathrm{T})^2\mathrm{S})$$

where ε_{eff} is an effective dielectric constant with a real part ε'_{eff} and an imaginary part ε''_{eff}. The dielectric constant for sand, clay and silt are $\varepsilon'_{sand}=3$, $\varepsilon'_{silt},\varepsilon'_{clay}=5$ and $\varepsilon''_{sand},\varepsilon''_{silt},,\varepsilon''_{clay}=0.078$. $\sigma_{25\,°C}$ is conductivity for saline water at $25\,°C$. φ is an empirical parameter. In short, the input parameters in the Park model include soil volumetric water content θ, soil temperature (T), percentages of sand and clay, organic matter content and soil salinity.

S2 The Local Ensemble Transform Kalman Filter (LETKF)

The derivation of the LETKF given in Hunt, et al. [5] is summed. Given an ensemble $\boldsymbol{x}_{t-1}^{a(i)}$ of m-dimensional model state vectors at time $t-1$, an nonlinear model $\boldsymbol{M}_{t-1,t}$ is applied to drive the evolution of each ensemble member to form a background ensemble $\boldsymbol{x}_t^{b(i)}$ at time t (equation (S2-1)). $\boldsymbol{y}_t^o$ is a vector of observations at time t. These observations are related to the state vector through an operattion operator $\boldsymbol{H}_t$ (equation (S2-2)).

$$\boldsymbol{x}_t^{b(i)}=\boldsymbol{M}_{t-1,t}\left(\boldsymbol{x}_{t-1}^{a(i)}\right)+\boldsymbol{\epsilon}_t \quad \text{(S2-1)}$$

$$\boldsymbol{y}_t^o=\boldsymbol{H}_t\left(\boldsymbol{x}_t^{b(i)}\right)+\boldsymbol{\varepsilon}_t \quad \text{(S2-2)}$$

where $i = 1,2 \dots. k$, with k as the ensemble member size. The subscripts $a(i)$ and $b(i)$ denote the analysis (posterior) and background (prior), respectively, of member i. $\boldsymbol{\epsilon}_t$ and $\boldsymbol{\varepsilon}_t$ denote the model and observation errors, respectively. $\boldsymbol{\varepsilon}_t$ are assumed to be unbiased Gaussian and uncorrelated errors in time with known covariance matrices $\boldsymbol{R}$. In the following, the operations and results at the posterior t are concentrated, and the subscript t is dropped. Regarding the prior state estimate and its covariance, the sample mean and covariance of the prior ensemble are used (equations (S2-3 and S2-4)).

$$\overline{\boldsymbol{x}}^b = k^{-1}\sum_{i=1}^{k}\boldsymbol{x}^{b(i)} \tag{S2-3}$$

$$\boldsymbol{P}^b = (k-1)^{-1}\sum_{i=1}^{k}(\boldsymbol{x}^{b(i)} - \overline{\boldsymbol{x}}^b)(\boldsymbol{x}^{b(i)} - \overline{\boldsymbol{x}}^b)^T = (k-1)^{-1}\boldsymbol{X}^b(\boldsymbol{X}^b)^T \tag{S2-4}$$

Formally, the LETKF aims to find the initial state and/or parameters that minimize the distance to the prior estimate, weighted by $\boldsymbol{P}^b$, while also minimizing the distance of the model trajectory to the observations, weighted by $\boldsymbol{R}^{-1}$ at time t. The Kalman filter cost function to be minimized to determine the posterior mean $\overline{\boldsymbol{x}}^a$ is formulated in equation (S2-5).

$$\boldsymbol{J}(\boldsymbol{x}) = \left(\boldsymbol{x} - \overline{\boldsymbol{x}}^b\right)^T\left(\boldsymbol{P}^b\right)^{-1}\left(\boldsymbol{x} - \overline{\boldsymbol{x}}^b\right) + [\boldsymbol{y}^o - \boldsymbol{H}(\boldsymbol{x})]^T\boldsymbol{R}^{-1}[\boldsymbol{y}^o - \boldsymbol{H}(\boldsymbol{x})] \tag{S2-5}$$

The $m \times m$ background covariance matrix $\boldsymbol{P}^b$ is noted to be invertible, as it exhibits a rank of at most $k - 1$. However, as a symmetric matrix, it is one-to-one on its column space S (i.e., the column space of $\boldsymbol{X}^b$) spanned by the prior ensemble perturbations. $\boldsymbol{P}^b$ and $\boldsymbol{J}(\boldsymbol{x})$ are therefore well defined on S, and minimization is carried out in this space. To obtain the posterior ensemble on S, a linear transformation is assumed for $\boldsymbol{X}^b$ from a k-dimensional space $\tilde{S}$ onto S, and local analysis is performed in $\tilde{S}$. Let $\boldsymbol{w}$ denote a vector in $\tilde{S}$, $\boldsymbol{X^b w}$ then belongs to the space S spanned by the prior ensemble perturbations, and $\boldsymbol{x} = \overline{\boldsymbol{x}}^b + \boldsymbol{X}^b\boldsymbol{w}$ is the corresponding model state. Let l be the number of scale observations used in the analysis. Assuming $\boldsymbol{w}$ is a Gaussian random vector with mean 0 and covariance $(k-1)^{-1}\boldsymbol{I}$, $\boldsymbol{x} = \overline{\boldsymbol{x}}^b + \boldsymbol{X}^b\boldsymbol{w}$ is Gaussian with mean $\overline{\boldsymbol{x}}^b$ and covariance $\boldsymbol{P}^b = (k-1)^{-1}\boldsymbol{X}^b(\boldsymbol{X}^b)^T$. Then, equation (S2-5) becomes equation (S2-6):

$$\tilde{\boldsymbol{J}}(\boldsymbol{w}) = (k-1)\boldsymbol{w}^{\boldsymbol{T}}\boldsymbol{w} + [\boldsymbol{y}^o - \boldsymbol{H}(\overline{\boldsymbol{x}}^b + \boldsymbol{X}^b\boldsymbol{w})]^T\boldsymbol{R}^{-1}[\boldsymbol{y}^o - \boldsymbol{H}(\overline{\boldsymbol{x}}^b + \boldsymbol{X}^b\boldsymbol{w})] \tag{S2-6}$$

An ensemble $\boldsymbol{y}^{b(i)} = \boldsymbol{H}(\boldsymbol{x}^{b(i)})$ of prior observation vectors is defined with mean $\overline{\boldsymbol{y}}^{b}$, and the $l \times k$ matrix $\boldsymbol{Y}^{b} = \boldsymbol{y}^{b(i)} - \overline{\boldsymbol{y}}^{b}$ is the perturbation matrix. The linear approximation is assumed by equation (S2-7):

$$\boldsymbol{H}(\overline{\boldsymbol{x}}^{b} + \boldsymbol{X}^{b}\boldsymbol{w}) \approx \overline{\boldsymbol{y}}^{b} + \boldsymbol{Y}^{b}\boldsymbol{w} \tag{S2-7}$$

Then the cost function (equation (S3-6)) yields a quadratic form and is formulated by equation (S2-8):

$$\tilde{\boldsymbol{J}}^{*}(\boldsymbol{w}) = (k-1)\boldsymbol{w}^{\boldsymbol{T}}\boldsymbol{w} + [\boldsymbol{y}^{o} - \overline{\boldsymbol{y}}^{b} - \boldsymbol{Y}^{b}\boldsymbol{w})]^{T}\boldsymbol{R}^{-1}[\boldsymbol{y}^{o} - \overline{\boldsymbol{y}}^{b} - \boldsymbol{Y}^{b}\boldsymbol{w}] \tag{S2-8}$$

This cost function (equation (S3-8)) occurs in the form of the Kalman filter cost function, using the prior mean $\overline{\boldsymbol{w}}^{b} = 0$, prior covariance $\widetilde{\boldsymbol{P}}^{b} = (k-1)^{-1}\boldsymbol{I}$ and $\boldsymbol{Y}^{b}$ acting as the observation operator. Then, analogous to the updates in the Kalman filter:

$$\overline{\boldsymbol{w}}^{a} = \widetilde{\boldsymbol{P}}^{\boldsymbol{a}}(\boldsymbol{Y}^{b})^{T}\boldsymbol{R}^{-1}(\boldsymbol{y}^{o} - \overline{\boldsymbol{y}}^{b}) \tag{S2-9}$$

$$\widetilde{\boldsymbol{P}}^{\boldsymbol{a}} = [(k-1)\boldsymbol{I} + (\boldsymbol{Y}^{b})^{T}\boldsymbol{R}^{-1}\boldsymbol{Y}^{\boldsymbol{b}}]^{-1} \tag{S2-10}$$

In model space, the posterior mean and covariance are formulated by equations (S2-11 and S2-12),

$$\overline{\boldsymbol{x}}^{a} = \overline{\boldsymbol{x}}^{b} + \boldsymbol{X}^{b}\overline{\boldsymbol{w}}^{a} \tag{S2-11}$$

$$\boldsymbol{P}^{a} = \boldsymbol{X}^{b}\widetilde{\boldsymbol{P}}^{a}(\boldsymbol{X}^{b})^{T} \tag{S2-12}$$

Then, the posterior ensemble described by $\overline{\boldsymbol{X}}^{a} = \boldsymbol{X}^{b}\boldsymbol{W}^{a}$ also must be updated, in which the symmetric square root is used to determine $\boldsymbol{W}^{a}$ based on $\widetilde{\boldsymbol{P}}^{\boldsymbol{a}}$.

$$\boldsymbol{W}^{a} = [(k-1)\widetilde{\boldsymbol{P}}^{\boldsymbol{a}}]^{1/2} \tag{S2-13}$$

Finally, $\overline{\boldsymbol{w}}^{a}$ is added to each column of $\boldsymbol{W}^{a}$ to form the vector $\boldsymbol{w}^{a(i)}$. $\boldsymbol{w}^{a(i)}$, as the weight vector, is used to obtain the posterior ensemble in the model space:

$$\boldsymbol{x}^{a(i)} = \overline{\boldsymbol{x}}^{b} + \boldsymbol{X}^{b}\boldsymbol{w}^{a(i)} \tag{S2-14}$$

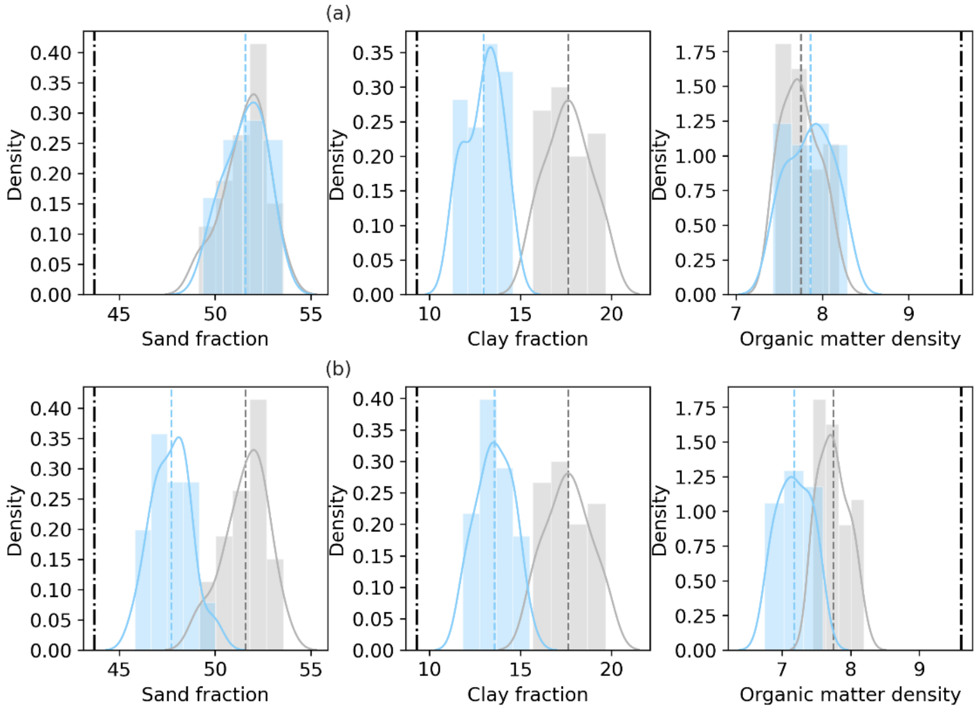

Figure S1. Prior and posterior distributions of sand fraction (%), clay fraction (%) and organic matter density (kg/m³) at the third layer (i.e., 11.89 cm), with the truth based on laboratory measurements of the 0–5 cm soil layer sampled in the field. The top panel labeled (a) displays the Only_Para experiment results based on SMAP T_B^H assimilation. The bottom panel (b) displays the Joint_Updt experiment results. In each subfigure, gray indicates the prior, light blue indicates the posterior, and the black dash-dotted line indicates the laboratory measurements.

Table S1. Retrieved soil properties at the third layer (i.e., 11.89 cm) and their calculated standard deviations (_std) from the experiment with only soil properties updated (Only_Para) and experiment with both soil properties and soil moisture estimates (Joint_Updt) by assimilating SMAP $\mathrm{T_B^H}$. z^b and z^a denote background and analysis, respectively.

Soil property	True	$\boldsymbol{z}^b$	$\boldsymbol{z}^b$_std	Only_Para		Joint_Updt	
				$\boldsymbol{z}^a$	$\boldsymbol{z}^a$_std	za	za_std
Sand fraction (%)	43.71	46.06	1.01	51.57	1.07	47.72	0.99

Clay fraction (%)	9.30	17.62	1.21	12.96	0.97	13.56	0.97
Organic matter density (kg/m³)	9.61	17.22	0.47	7.86	0.26	7.18	0.24

Table S2. RMSEs of the retrieved soil properties at the third layer (i.e., 11.89 cm) from the experiment with only soil properties updated (Only_Para) and experiment with both soil properties and soil moisture estimates (Joint_Updt) by assimilating SMAP T_B^H.

Soil property	$\boldsymbol{z}^b$_RMSE	Only_Para		Joint_Updt	
		$\boldsymbol{z}^a$_RMSE	Reduction	za_RMSE	Reduction
Sand fraction (%)	7.96	7.93	0.4%	4.13	48.1%
Clay fraction (%)	8.4	3.79	54.8%	4.37	48.0%
Organic matter density (kg/m³)	1.87	1.77	5.3%	2.44	Ineffective

Reference

1. Park, C.-H.; Behrendt, A.; LeDrew, E.; Wulfmeyer, V., New Approach for Calculating the Effective Dielectric Constant of the Moist Soil for Microwaves. *Remote. Sens.* **2017,** 9, 732.
2. Brown, W. F., Dielectrics. In *Dielectrics*, Springer: Berlin, Germany, 1956; Vol. 17, pp 1-154.
3. Park, C. H.; Montzka, C.; Jagdhuber, T.; Jonard, F.; De Lannoy, G.; Hong, J.; Jackson, T. J.; Wulfmeyer, V., A Dielectric Mixing Model Accounting for Soil Organic Matter. *V. Z. J.* **2019,** 18, 190036.
4. Tóth, B.; Weynants, M.; Nemes, A.; Makó, A.; Bilas, G.; Tóth, G., New generation of hydraulic pedotransfer functions for Europe. *Eur. J. Soil. Sci.* **2015,** 66, 226-238.
5. Hunt, B. R.; Kostelich, E. J.; Szunyogh, I., Efficient data assimilation for spatiotemporal chaos: A local ensemble transform Kalman filter. *Physica D: Nonlinear Phenomena* **2007,** 230, 112-126.
